# Juvenile Justice—Translational Research on Interventions for Adolescents in the Legal System (JJ-TRIALS): a cluster randomized trial targeting system-wide improvement in substance use services

**DOI:** 10.1186/s13012-016-0423-5

**Published:** 2016-04-29

**Authors:** Danica K. Knight, Steven Belenko, Tisha Wiley, Angela A. Robertson, Nancy Arrigona, Michael Dennis, John P. Bartkowski, Larkin S. McReynolds, Jennifer E. Becan, Hannah K. Knudsen, Gail A. Wasserman, Eve Rose, Ralph DiClemente, Carl Leukefeld, the JJ-TRIALS Cooperative

**Affiliations:** 1 Institute of Behavioral Research, Texas Christian University, Fort Worth, TX USA; 2 Department of Criminal Justice, Temple University, Philadelphia, PA USA; 3 National Institute on Drug Abuse, Bethseda, MD USA; 4 Social Science Research Center, Mississippi State University, Starkville, MS USA; 5 Council of State Governments Justice Center, Austin, TX USA; 6 Chestnut Health Systems, Normal, IL USA; 7 Department of Sociology, University of Texas at San Antonio, San Antonio, TX USA; 8 Center for the Promotion of Mental Health in Juvenile Justice, Columbia University/NYSPI, New York, NY USA; 9 Behavioral Science, University of Kentucky, Lexington, KY USA; 10 Rollins School of Public Health, Emory University, Atlanta, GA USA

**Keywords:** Evidence-based practice implementation, System change, Interagency collaboration, Substance use, Treatment services, Data-driven decision-making, Juvenile justice, Adolescent, Justice-involved youth, Cluster randomized trial

## Abstract

**Background:**

The purpose of this paper is to describe the Juvenile Justice—Translational Research on Interventions for Adolescents in the Legal System (JJ-TRIALS) study, a cooperative implementation science initiative involving the National Institute on Drug Abuse, six research centers, a coordinating center, and Juvenile Justice Partners representing seven US states. While the pooling of resources across centers enables a robust implementation study design involving 36 juvenile justice agencies and their behavioral health partner agencies, co-producing a study protocol that has potential to advance implementation science, meets the needs of all constituencies (funding agency, researchers, partners, study sites), and can be implemented with fidelity across the cooperative can be challenging. This paper describes (a) the study background and rationale, including the juvenile justice context and best practices for substance use disorders, (b) the selection and use of an implementation science framework to guide study design and inform selection of implementation components, and (c) the specific study design elements, including research questions, implementation interventions, measurement, and analytic plan.

**Methods/design:**

The JJ-TRIALS primary study uses a head-to-head cluster randomized trial with a phased rollout to evaluate the differential effectiveness of two conditions (Core and Enhanced) in 36 sites located in seven states. A *Core* strategy for promoting change is compared to an *Enhanced* strategy that incorporates all core strategies plus active facilitation. Target outcomes include improvements in evidence-based screening, assessment, and linkage to substance use treatment.

**Discussion:**

Contributions to implementation science are discussed as well as challenges associated with designing and deploying a complex, collaborative project.

**Trial registration:**

NCT02672150.

## Background

Substance use is common among adolescent offenders and relates to delinquency, psychopathology, social problems, risky sex and sexually transmitted infections like HIV, and other health problems [1, 2]. An estimated 70 % of arrested juveniles have had prior drug involvement [3] and over 1/3 have substance use disorders [4, 5]. Arrested youth initiate substance use earlier than other adolescents, leading to more problematic substance use and higher recidivism [6–8].

US juvenile courts processed 1,058,500 delinquency cases in 2013, with 31 % of cases adjudicated [9]. Most youth who come into contact with the juvenile justice (JJ) system are supervised in the community [10], and the proportion of youth under community supervision is increasing as states across the country seek alternatives to incarceration/detention [9, 11, 12]. Given the contribution of substance use to recidivism, JJ agencies are uniquely positioned to significantly impact public health through substance use identification and early intervention [13].

Because substance use services are generally provided outside the JJ system [14], cross-system linkage is necessary, but often problematic [15–17]. Even when linkages are in place, some community service providers do not consistently offer evidence-based services [18]. Collaboration requires communication across agencies that have historically existed as silos, with distinct cultures and belief systems about the effectiveness and importance of substance use treatment [19–21]. This context offers an ideal opportunity for implementation science, as communities strive to better meet the needs of youth.

### The JJ-TRIALS Cooperative

The Juvenile Justice—Translational Research on Interventions for Adolescents in the Legal System (JJ-TRIALS) is a cooperative research initiative funded by the National Institute on Drug Abuse (NIDA). Six research centers (RCs: Columbia University, Emory University, Mississippi State University, Temple University, Texas Christian University, University of Kentucky) and one coordinating center (CC: Chestnut Health Systems) were funded in July 2013. Each RC recruited one or more JJ Partners to participate in all planning and implementation activities from the outset. The JJ-TRIALS steering committee (SC: composed of principal investigators, JJ Partners, and a NIDA project scientist) was charged by NIDA with developing a study protocol that achieved two goals: (1) improving the delivery of evidence-based practices (EBPs) in community-based JJ settings and (2) advancing implementation science.

Collaboration and cooperation among JJ-TRIALS researchers, partners, and NIDA personnel are critical for study protocol development, refinement, adherence, and implementation. Each of these constituencies provides input on feasibility, utility, and scientific rigor. This approach ensures a study design that meets scientific and partner expectations, while also keeping feasibility in focus. JJ partners provide a real-world comprehensive understanding of the JJ system and its processes through study development, thus assuring a meaningful focus and increasing the study’s potential impact.

### Developing the study protocol

The Study Design Workgroup focused on five goals during the development of the JJ-TRIALS protocol: (1) conceptualizing how substance use should be addressed through partnerships between JJ and behavioral health (BH) agencies, (2) identifying evidence-based tools for addressing substance use, (3) identifying a conceptual framework to understand the process of implementing changes, (4) using that framework to guide overall study design, and (5) testing two distinct strategies for implementing desired changes. The final study protocol conforms to a hybrid implementation design [22]. It examines organizational-level implementation outcomes and youth outcomes, using a mixed-methods approach [23]. Primary aims are to (1) improve the continuum of substance use services for juvenile offenders under community supervision and (2) test the effectiveness of two implementation strategies for promoting system-wide change.

### The guiding evidence-based practices framework

Best practices for substance use treatment involve a logically sequenced continuum ranging from initial screening to placement and retention in appropriate care. The JJ-TRIALS Cooperative sought to specify how screening, assessment, service referral, and treatment services are interconnected in the identification and linkage to care. The design team developed a service cascade framework that captured the receipt of BH services and provided a unifying approach to guide site activities and study outcomes across a diverse set of sites with unique needs and goals.

The JJ-TRIALS Behavioral Health Services Cascade (hereinafter the Cascade) was modeled after the HIV care cascade, a widely used framework for depicting both gaps in HIV surveillance and treatment [24–26]. The Cascade provides a data-driven framework for understanding how justice-involved youth move from JJ to community-based BH providers as substance use problems are identified and responses implemented. The Cascade is premised on the idea that the overlap between substance use problems and JJ contact necessitates screening of all youth who come into contact with the justice system [27, 28]. In an ideal system, a positive initial screen would lead to a more in-depth assessment and, if warranted, subsequent linkage to evidence-based care in the community. There are numerous evidence-based screening and assessment instruments [29, 30], various evidence-based treatment and prevention interventions [31], and promising interventions for linking youth to community-based providers [32, 33].

**Fig. 1:**
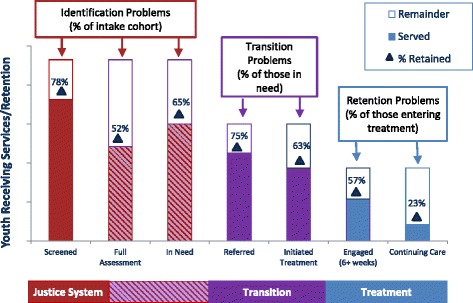
Hypothetical retention in the Cascade as youth transition across service systems

Evidence shows that the service continuum begins to break down at the initial step of screening in most JJ settings. A national survey of juvenile probation agencies revealed that only 47.6 % reported using a standardized tool to screen and/or assess substance use [17]. Furthermore, a typical specialty adolescent substance use treatment program only adopts about half of high-quality substance use care indicators and EBPs [34]. Figure 1 represents hypothetical data for the Cascade as youth transition across service systems, with each column representing the difference between ideal and actual levels of service delivery. Differences between ideal and actual levels represent problems related to identification, transition, and retention in care. Youth with substance use problems can only be engaged in appropriate treatment if their needs are identified.

Although the Cascade serves as a framework for setting goals around improved evidence-based practice, the study protocol allows sites to choose where on the Cascade they will focus their improvement efforts. This degree of agency-level autonomy recognizes that different EBPs will “fit” better across different agencies (i.e., address the needs of youth, work within constraints of the system). Each agency, informed by data and best practices, sets its own goals for reducing service gaps. The study protocol uses a series of menus of evidence-based screening and assessment tools and treatments to help guide these decisions, but does not dictate that sites focus on a specific point on the Cascade or a particular EBP.

### The guiding implementation science framework

The Exploration, Preparation, Implementation, Sustainment (EPIS) framework of Aarons and colleagues guides the design of this study [35]. Consistent with models of quality improvement in healthcare systems [36], EPIS considers the multilevel nature of service systems, the organizations within systems, and client needs during the process of implementing a new intervention. The EPIS model posits four phases of organizational processes during system change. The *Exploration Phase* involves identification of the problem, appropriate evidence-based solutions, and factors that might impact implementation. Once a proposed solution is identified for adoption, the *Preparation Phase* begins. This phase involves bringing together stakeholders in a planning process [37], which can be complex, depending on the number of stakeholders and potentially competing priorities and needs [38]. The *Implementation Phase* begins when initiating change-related activities. Factors affecting implementation include outer context political and funding concerns, inner organizational context issues (e.g., fit with clinician productivity and work demands), and consumer concerns (e.g., applicability of practices for client needs) [39]. When the new practice is routinely used, the *Sustainment Phase* begins. Sustainment may be facilitated by the degree to which the new services or changes are institutionalized at different levels in the service setting (i.e., system, organizations).

The Cooperative has adapted EPIS to address the complex context within which the JJ-TRIALS study occurs. First, EPIS has typically been applied to the implementation and adoption of one specific EBP [40]. In JJ-TRIALS, sites are asked to select a target goal from the Cascade and implement an EBP that addresses that goal. Thus, each study site could potentially implement a different EBP. Second, while the linear nature of EPIS guides the general design (timing of implementation strategies and measurement), it also implies a dynamic process. In the current study, sites are taught to use data to inform implementation decisions through the application of rapid-cycle testing [41–43]. With each “test,” there are subsequent periods of exploration (e.g., what worked, what went wrong), preparation (e.g., modifications to the original plan), and implementation (e.g., enacting the revised plan). JJ-TRIALS is designed to capture these activities to explore and refine the EPIS model.

## Methods/Design

### Selecting the implementation interventions

Implementation studies typically have focused on a single evidence-based intervention [44–46], a specific set of best practices [47, 48], generic best practices [49], or a single evidence-based instrument [50]. Few studies have focused on outcomes that cross service system sectors of care [44]. Head-to-head organizational comparative effectiveness trials are rare, in part because the resources needed to execute them often exceed those available in a typical National Institutes of Health (NIH) grant. In JJ-TRIALS, several discrete implementation strategies were combined and manualized to address organizational and system barriers [51]. This effort leverages the resources of RCs with the practical guidance of JJ partners to field a multisite, direct comparison of implementation strategies in a relatively large sample of sites.

The JJ-TRIALS protocol compares two novel implementation interventions that combine several implementation strategies with demonstrated efficacy. These strategies include a needs assessment [52], ongoing training and education [37, 53], local change teams with facilitation [54, 55], and data-driven decision-making [56, 57]. The basic implementation approach compares a *Core* set of intervention strategies to a more *Enhanced* set that incorporates all core components plus active facilitation. Across both study conditions, data-driven decision-making serves as a common thread.

#### Data-driven decision-making (DDDM)

According to the JJ-TRIALS partners, most JJ departments are encouraged to use data to inform decisions, yet few JJ agencies are adequately skilled and resourced in doing so. A number of recent JJ initiatives such as the MacArthur Foundation’s Models for Change [58] have emphasized the importance of making data-informed policy choices. Focusing on systematic data collection, synthesis, and interpretation can help agencies to transform the ways they address problems and implement future change. In design discussions, JJ partners questioned whether providing tools and training would be sufficient or whether a guided “mentoring” approach would be needed to enact system-wide change using DDDM.

DDDM is the process by which key stakeholders collect, analyze, and interpret data to guide efforts to refine or reform a range of outcomes and practices [59]. In JJ settings, DDDM has been used to guide system-wide reform to reduce recidivism and system costs while improving related outcomes such as public safety and access to evidence-based services [60–62]. In one instance, DDDM was associated with a 5-year doubling of the proportion of youth accessing EBPs while reducing arrest rates by almost half [58]. This approach has the potential to address unmet substance use treatment needs for JJ-involved youth.

#### Implementation intervention components

**Fig. 2:**
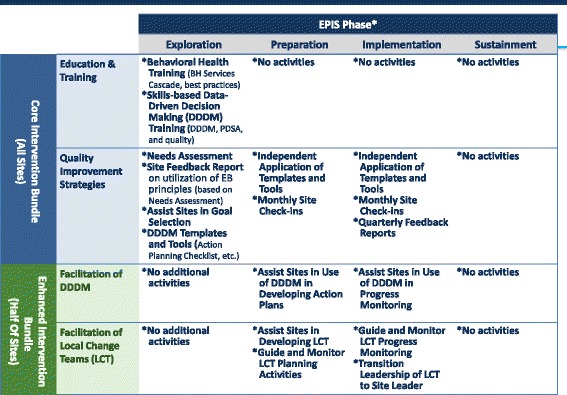
Selection and timing of Core and Enhanced components

The two sets of implementation intervention strategies tested in JJ-TRIALS are additive (see Table 1 for a description of Core and Enhanced components). The *Core* condition includes five interventions implemented at all sites during the 6-month baseline period (see timeline below): (1) JJ-TRIALS orientation meetings, (2) needs assessment/system mapping, (3) behavioral health training, (4) site feedback report, and (5) goal achievement training. Following the baseline period, two additional Core components are delivered to all sites: (6) monthly site check-ins and (7) quarterly reports. As part of goal achievement training, sites receive assistance in using their site feedback report to select goals to meet their local needs. Sites are trained on using data to inform decisions (e.g., selecting a goal, applying plan-do-study-act) and enlisting DDDM templates and tools (developed as part of the project) to plan and implement proposed changes. While DDDM principles are expected to facilitate change, organizations may need additional support to apply these principles to their improvement efforts during the implementation phase. The *Enhanced* condition adds continuing support for the use of DDDM tools by adding research staff facilitation of DDDM over 12 months and formalized local change teams (LCTs) featuring representation from the JJ agency and a local BH provider (with meetings facilitated by research staff). Figure 2 depicts how selection and timing of specific components was informed by EPIS.

**Table 1:** Description of Core and Enhanced intervention components

Intervention component	Study condition	Description	Timing	Participant investment
Leadership orientation	Core	RC meets with site leadership to describe overall project aims and activities (including the Cascade).	Month 1	1 h
Needs assessment: systems mapping exercise/focus group interview	Core	RC meets with site inter-agency workgroup to (a) create a systems map of case-processing in their jurisdiction depicting “linkages” with community behavioral health partners and (b) explore strengths and limitations of local practices corresponding to each step in the Cascade.	Month 3 (baseline)	1 h for systems mapping exercise and 2 h for focus group
			Month 21 (post-experiment)	
Line staff orientation	Core	Almost identical to the leadership orientation described above, with more emphasis on staff participation and expectations.	Month 5 (baseline)	1 h
Site feedback report	Core	Transcripts from the needs assessment discussion, data from site’s management information system, and National Survey responses form the basis of the site feedback report. RC presents the 25-page site feedback report to site inter-agency workgroup.	Month 7 (baseline)	1 h
			Month 26 (project end)	
Behavioral health training: *Charting a Course to Care*	Core	Participants participate in four self-paced online didactic tutorials, plus four live skills supervision sessions delivered online via webinar.		
Module 1 (online)		Substance use and correlated conditions:	Month 6 (baseline)	45 min, self-paced
		Reviews co-occurring conditions that go along with adolescent substance use, prevention programs and their importance in adolescent SU, STI and HIV risks for adolescent SU, and the importance of integrated treatment.		
Module 2 (online)		Behavioral Health Cascade:	Month 6 (baseline)	45 min, self-paced
		Reviews the Cascade, how it can be used to identify strengths and targets for change, common breakdowns in agency communication, and how EB instruments and programs can be used.		
Module 3 (online)		Family engagement:	Month 6 (baseline)	45 min, self-paced
		Focuses on strategies to engage parents, or other caregivers and adolescents as they access behavioral health services, communicating to the parents about JJ recommendations for their child and why this care is important (to help secure their active participation or “buy-in”).		
Module 4 (online)		Case planning across agencies:	Month 6 (baseline)	45 min, self-paced
		Reviews strategies for sharing expectations and information with behavioral health partners, understand the rationale behind agency accreditation, and learn how to identify the best behavioral health agencies and providers in the area.		
Live activity 1 (webinar)		Family engagement:	Month 7 (baseline)	2. 1-h sessions
		Live supervision format that builds on information from unit 3 helping JJ to develop skills to share screening results and evidence that gets the family to commit to initiating and engaging in treatment services.		
Live activity 2 (webinar)		Case planning across agencies:	Month 7 (baseline)	2. 1-h sessions
		Live supervision format that builds on information from unit 4 and discusses tools, techniques, and communication practices (to share information across agencies and increase linkage to treatment).		
Goal achievement training	Core	Onsite training where members of the inter-agency workgroup learn about and select a “goal” to work on given the information contained in their site feedback report. Following goal selection, teams are trained on how to review and use data to inform the decisions they make during efforts to improve identification of substance use needs and linkage to services (e.g., plan-do-study-act and SMART goal activities).	Month 7 (baseline)	6 h over 1 or 2 days
Part 1: goal selection support				
Part 2: data-driven decision-making				
Monthly site check-ins	Core	Telephone conversation with the site liaison about activities toward their selected goal in the previous month	Months 8–19 (experiment)	9 h over 12 months
Quarterly reports	Core and Enhanced	RC generates quarterly reports that are emailed to the site liaison. The report presents data on retention in the Cascade (screening, assessment, referral, treatment initiation, treatment engagement, continuing care) at 3-month intervals.	Months 11, 13, 15, and 17 (experiment)	2 h over 12 months
Interagency workgroup meetings	Core	Periodic meetings to work toward achieving the selected goal using PDSA and other strategies learned through the goal achievement training	Months 8–19 (experiment)	At sites’ discretion
Facilitator-local champion calls/meetings	Enhanced	Calls and meetings (between RC and site) designed to (1) prepare for upcoming local change team (LCT) meetings, (2) debrief from previous LCT meetings, and (3) transition from external facilitator to internal facilitator	Months 8–19 (experiment)	6–24 h total; projected average of 15 h over 12 months
Local change team (LCT) meetings	Enhanced	Monthly meetings principally organized around the following activities: goal selection; plan phase of PDSA; study phase of PDSA; strategies for acquisition, utilization, and interpretation of agency data; and sustainment of new practices	Months 8–19 (experiment)	14–21 h total over 12 months
LCT transition to sustainment meeting	Enhanced	Single close-out meeting as facilitation ends (note: not formally called “close-out” to avoid undermining sustainment)	Month 19 (at conclusion of experiment)	1.5 h
Study close-out meeting	Core and Enhanced	RC meets with site inter-agency workgroup (or LCT in Enhanced) at project end	Month 26 (at conclusion of sustainment)	1.5 h

### Study design

**Fig. 3:**
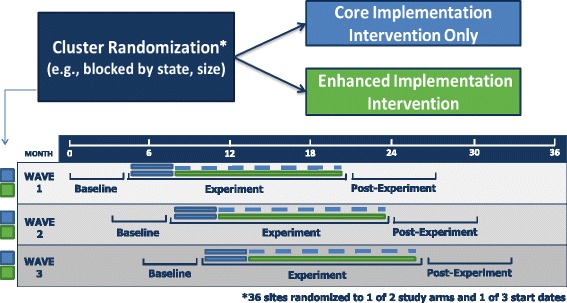
JJ-TRIALS Study Design

The design uses a cluster randomized trial with a phased rollout to evaluate the differential effectiveness of the Core and Enhanced conditions in 36 sites (18 matched pairs—see below) in 7 states. The design features randomization to one of two conditions, randomization to one of three cohorts (with start times spaced 2 months apart), the inclusion of a baseline period in both experimental conditions, and data collection at regular intervals (enabling time series analyses; see Fig. 3). In addition to comparing the two implementation conditions, it also allows sites to serve as their own controls by using an interrupted time series design with the baseline period as an existing practice control. This design enables three time-series comparisons: (1) Baseline (“activities as usual”) versus Core, (2) Baseline versus Enhanced, and (3) Core versus Enhanced.

Primary research questions include:

1. Does the Core and/or Enhanced intervention reduce unmet need by increasing Cascade retention related to screening, assessment, treatment initiation, engagement, and continuing care?
2. Does the addition of the Enhanced intervention components further increase the percentage of youth retained in the Cascade relative to the Core components?
3. Does the addition of the Enhanced intervention components improve service quality relative to Core sites?
4. Do staff perceptions of the value of best practices increase over time, and are increases more pronounced in Enhanced sites?

The study also includes exploratory research questions. Examples include: How do sites progress through EPIS phases with and without facilitation? Are Enhanced sites more successful in implementing their chosen action plans, achieving greater improvement in cross-systems interagency collaboration, and experiencing greater reductions in 1-year recidivism rates? Is one condition more cost-effective than the other? And how do inner and outer context measures (e.g., system, organizational, staff) moderate relationships between experimental conditions and service outcomes?

#### Sample

The sample includes 36 sites, with each site composed of one JJ and one or two BH agencies (overall more than 72 participating organizations). Sites were matched into pairs within state systems (based on local population, number of youth referred to JJ, number of staff, and whether EBPs are used). JJ agencies include probation departments (in six states) or drug courts (in one state); BH providers include substance use treatment providers within a county or service region. JJ inclusion criteria were (a) ability to provide youth service records, (b) serve youth under community supervision, (c) access to treatment provider(s) if treatment is not provided directly, (d) minimum average case flow of 10 youth per month, (e) minimum of 10 staff per site, and (f) a senior JJ staff member who agrees to serve as site leader/liaison during the study. Study sites are geographically dispersed and were identified by state JJ agencies (and not selected for particular substance use or related BH service needs).

At the beginning of the project, each site forms an *Interagency Workgroup* composed of 8–10 representatives from JJ and BH agencies. Recommended composition includes representatives of JJ leadership (e.g., Chief Probation Officer), BH leadership (e.g., Program Director), other JJ and BH agency staff, and other key stakeholders likely to be involved in improvement efforts (e.g., Juvenile Court Administrator, JJ Data Manager).

At least 360 staff members from participating JJ and BH agencies are expected to participate in one or more study activities. Information from at least 120 individual youth case records per site is de-identified and extracted from site data files on a quarterly basis throughout the study period (a minimum sample of 4320 de-identified service records). Interagency workgroup participation, staff survey responses, and youth records are nested within sites.

#### Recruitment and consenting

Partners facilitated identification and recruitment of JJ agencies. RC staff described study involvement and worked with JJ leadership to identify and recruit the BH partner. JJ agency leadership provided signed letters of commitment and, if required by agency policy or state law, court orders authorizing RC access to confidential juvenile case records. Individual staff recruitment occurs immediately after each leadership and line staff orientation meeting. During orientations, all aspects of the research study are explained and informed consent is obtained from participants, consistent with institutional review board (IRB) protocols at each RC.

#### Randomization

The design features two stages of randomization: (a) to one of three start times as part of a phased rollout and (b) to the Core or Enhanced condition. The CC was responsible for all randomization procedures. For the first stage, RCs were used as strata and the six county sites within each were matched into pairs in terms of the number of youth (ages 10–19) in the county based on the 2010 census, the number of youth entering community supervision, the number of staff in community supervision, whether they used standardized screeners/assessments and evidence-based treatment. Each RC PI reviewed matches and ensured comparability prior to randomization. Within each RC, the three resulting pairs were then randomly assigned to one of three start times using a random number generator in Excel. This procedure was utilized to smooth out the logistical burden of implementation and to control for the influence of other exogenous factors [63, 64].

For the second stage of randomization, one site in each pair was randomly assigned to Core and the other to Enhanced. Given that there were only 18 pairs of sites, “optimal” randomization was used to find the most balanced pattern of assignment across each RC. This approach involved running 10,000 permutations of the possible assignments of sites within each pair to condition. For each of these, multivariate Hotelling’s *T* ^2^ was computed to assess the degree of balance on cohort and condition both within and across all RCs. The final randomization design was selected from a pool of the top 2 % of permutations balancing across all characteristics.

The study is also double-blinded such that neither the RC staff nor any county site staff are aware of their assignment until after both sites in a pair have completed the Core components. Once completed, the condition of both sites is revealed by the CC PI to RC PI and, subsequently, to sites. This design aspect is ideal in studies with multiple sites that have initial variability and require intensive researcher-driven activities such as training, monitoring, or coaching.

#### Power

For service record level hypotheses, 2160 bi-weekly observations are expected on service delivery outcome measures (36 sites × 60 bi-weekly periods). For site-level hypotheses, 72 observations are expected (36 sites × 2 data collection points), and for staff level hypotheses, a minimum of 1440 observations are expected, with 720 per condition (average of 10 staff × 36 sites × 4 time points). The effective *n* for power calculations in repeated measures analysis varies between a lower bound of the number of unique sites (*N* = 36) and an upper bound of the observations per condition (*O* = 1440 staff surveys or 2160 bi-weekly youth record periods), as a function of the Intraclass Correlation Coefficient (ICC) associated with the outcome measure (e.g., number of youth entering treatment) over time and the number of repeated measures per site. Assuming that the ICC is low (.2 or less), effect sizes in the small to moderate range (.25 to .35) should be detected with 80 % or more power [65]. Several strategies are employed to further increase power: (a) optimal randomization to evenly distribute the 36 sites as much as possible across start up time and condition, (b) using standardized measures to reduce measurement error, and (c) modeling site differences as a function of staff and organizational covariates.

### Measurement

A multilevel approach to measurement is necessary for our understanding of change processes within complex service systems [36, 66]. Youth interact with JJ agency staff who work within a larger organization; in turn, the organization operates within a system that includes BH providers, oversight agencies, and funders. The proposed measurement plan assesses information from these levels.

**Fig. 4:**
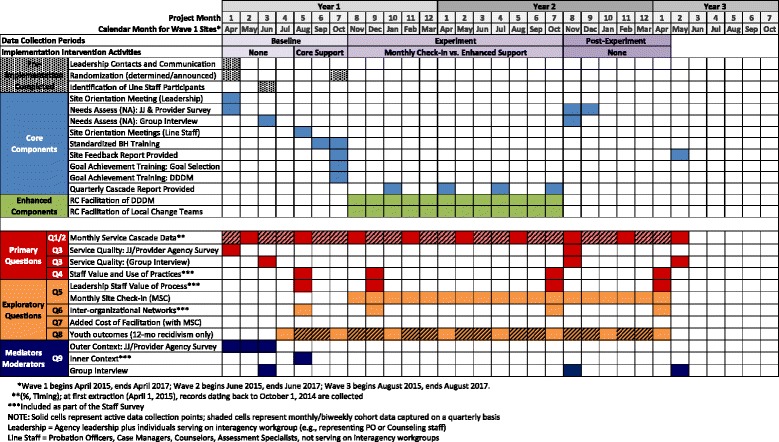
Timeline depicting intervention components (*top portion*) and data collection (*bottom portion*) for sites in wave 1

The design employs three data collection periods: baseline (6 months; generally corresponding to EPIS’ Exploration and Preparation phases), experiment (12 months; corresponding to EPIS’ Implementation phase), and post-experiment (6 months; corresponding to EPIS Sustainment phase). Figure 4 includes a timeline depicting all intervention components (top portion) and data collection (bottom portion) for sites in wave 1. During baseline, RCs initiate collection of de-identified youth records data related to the Cascade dating back to October 1, 2014, administer agency surveys, conduct a local needs assessment (systems mapping exercise and group interview with interagency workgroup members), and administer leadership and line staff surveys at participating agencies. Leadership and line staff complete follow-up surveys during months 2 and 12 of the experiment period and again at month 6 of the post-experiment period. A representative from each site reports progress toward site-selected goals (i.e., implementation activities) during a monthly site check-in phone call. In the Enhanced condition, local change team members complete implementation process surveys during the experiment period. The 6-month post-experiment period consists only of data collection, including youth record extraction, agency and staff surveys, group interview (to determine whether sites sustain new practices), and monthly site check-in calls. Data collection components are summarized in Table 2.

**Table 2:** Data collection components

Instrument	Description	Timing	Participant investment
START UP			
Pre-implementation checklist	Documents, orientations, memos of understanding, necessary approvals.	Month 1 (baseline)	Research staff; 1 h
INTERVENTION ACTIVITIES			
Core components			
Needs assessment group interview	Staff focus group (audio recorded and transcribed) designed to explore existing services along the Cascade, identification of strengths and gaps in services, creation of systems map; documentation of inner and outer context	Month 3 (baseline), month 21 (post-experiment)	Research staff; interagency workgroup (JJ and BH staff); 3 h
Site feedback report fidelity checklist	Documents the degree to which the intervention steps are followed and training content is covered	Month 7 (baseline), month 26 (project end)	Research staff; 10 min
Behavioral health training pre-post-test	Survey on the acquisition of knowledge gathered during behavioral health training sessions. Questions are asked before and immediately after each module	Month 6 (baseline)	JJ and BH staff; 5 min per module
Behavioral health training—live sessions fidelity checklist	Documents the degree to which the intervention steps are followed and training content is covered	Month 7 (baseline)	Research staff; 10 min per session
Goal achievement training fidelity checklist	Documents the degree to which the intervention steps are followed and training content is covered	Month 7 (baseline)	Research staff; 10 min
Goal achievement training pre-post-test	Survey of best practices for determining and defining goals, identification of site-selected goals along the Cascade, strategies for measuring progress toward goals, strategies for implementing and sustaining change	Month 7 (baseline)	JJ and BH staff; 10 min
Enhanced components (Enhanced condition only)			
Pre-implementation fidelity checklist	Documents the degree to which preparations for facilitation are followed	Month 8 (experiment)	RC facilitator; 10 min
Local change team (LCT) start-up fidelity checklist	Documents the degree to which plans for establishing LCTs and initiating facilitation are followed	Month 8 (experiment)	RC facilitator; 10 min
Meeting level facilitator reflection fidelity checklist (RC facilitator and local champion versions)	Documents the degree to which the facilitation protocol is followed and LCT discussions/progress toward goals	Month 8-19 (experiment)	RC Facilitator and Local Champion; 10 minutes per meeting
LCT fidelity checklist	Survey of LCT members regarding facilitation of their meetings	Months 8, 13, and 19 (experiment)	LCT; 10 min
Close-out meeting fidelity checklist	Documents the degree to which the close-out meeting plans are followed (including study debriefing)	Month 26 (at conclusion of post-experiment)	Research staff; 10 min
DATA COLLECTION			
Extraction of youth service records			
Community supervision survey	A subset of the JJ-TRIALS National Survey items (JJ version) used to elicit site characteristics and information about services provided	Month 1 (baseline), month 20 (sustainment)	JJ agency leadership; 2 h
Service provider survey	A subset of the JJ-TRIALS National Survey items (service provider version) used to elicit site characteristics and information about services provided	Month 1 (baseline), month 20 (sustainment)	BH agency leadership
Monthly site check-in form	Documents site activities that reflect progress toward achieving selected goals, collect information about process improvement efforts, and monitor changes to services based on data systems, staffing, and policy modifications	Months 8–25 (experiment, post-experiment)	Research staff interview site liaison; 30 min, monthly
Staff survey	The survey asks individuals about attitudes toward their workplace, services their agency provides, relationships between JJ and BH agencies in providing services, perceived value and use of substance use services (specific strategies for screening, assessment, referral, and treatment), and perceived value of HIV-STI and substance use prevention	Month 5 (baseline), months 9 and 19 (experiment), month 25 (post-experiment)	JJ and BH staff; 45 min, each administration

#### Fidelity

The JJ-TRIALS cooperative seeks to manage fidelity by balancing adherence to central elements of the implementation interventions and timely submission of research data with flexibility in addressing diverse site needs. This approach to fidelity aims to address the domains described by Proctor and colleagues [67] with regard to protocol adherence, dose/exposure, and quality. Protocol adherence is fostered by the provision of pre-implementation training activities to key principals (e.g., facilitators) along with the review of critical resources (e.g., detailed instructional manuals, preparation checklists). As implementation ensues, fidelity is further measured by RC-level reporting of the actual date of each study activity relative to its targeted completion date. The Timeline Compliance system tracks key elements of dose, such as the number of attendees at specific trainings [44]. Each implementation intervention has fidelity procedures that provide additional detail regarding adherence, dose, and quality. Procedures include automated reporting (e.g., online BH training sessions), observational ratings (e.g., webinar BH training sessions), facilitator-reported fidelity ratings (e.g., goal achievement training), and participant ratings (e.g., local change team meetings).

### Hypotheses

Table 3 summarizes the primary hypotheses corresponding to the research questions above. H1 and H2 focus on retention in the Cascade: H1 compares both experimental conditions to their respective baseline period, whereas H2 compares the differential effectiveness of Core versus Enhanced sites. Table 3 shows the working definition and formula for the rates of each step within the Cascade (see Fig. 1), designed to map onto existing and widely used performance metrics systems (Center for Substance Abuse Treatment adolescent treatment branch and National Outcome Monitoring System; the Network for the Improvement of Addiction Treatment (NIATx); the National Quality Forum; the Office of National Coordinator of Healthcare Reform; and Washington Circle Group). The rates shown are proportions of youth receiving the service within each site, divided by the number in the earlier step, with dashed lines highlighting changes in the denominator.

**Table 3:** JJ-TRIALS research questions and hypotheses

Primary research question	Corresponding hypotheses
1. Does the Core and/or Enhanced intervention reduce unmet need by increasing service cascade retention related to screening, assessment, treatment initiation, engagement and continuing care?	*H1:* Compared to baseline period, the percentage of *youth retained in the service cascade* will increase during the experiment period (in terms of % screened, % assessed, % in need, % referred, % initiated, % engaged, and % continuing care).
2. Does the addition of the Enhanced intervention components further increase the percentage of youth retained in the service cascade relative to the Core components?	*H2:* Compared to the Core intervention sites, the Enhanced Intervention sites will have greater improvements (relative to baseline) during the experiment period in their percentage of *youth retained in the service cascade* (in terms of % screened, % assessed, % in need, % referred, % initiated, % engaged, and % continuing care).
3. Does the addition of the Enhanced intervention components improve service quality relative to Core sites?	*H3:* Compared to the Core intervention sites, the Enhanced intervention sites will have greater improvements (relative to baseline) during the experiment period in service quality in terms of:
	a) *timing* (e.g., shortened length of time between arrest and screen),
	b) *content* (e.g., increased use of EBP to screen), or
	c) *procedure* (e.g., increased number of staff trained on using an EBP screener).
	(NOTE: H3b & H3c are largely descriptive and exploratory.)
4. Do staff perceptions of the value of best practices increase over time, and are increases more pronounced in enhanced sites? [staff within sites]	*H4:*
	*a)* Relative to Staff at the Core sites, staff at Enhanced sites will show a greater increase between baseline and end of experimental period in the perceived “value” and reported use of *evidence-based substance use treatment services* along the Cascade.
	b) Staff in both Core and Enhanced sites will show an increase between baseline and end of core support period in the perceived “value” of *HIV/STI prevention, testing, and treatment services*.
	c) Staff in both Core and Enhanced sites will show an increase between baseline and end of core support period in the perceived “value” of *evidence-based substance use prevention services*.

Latent Growth Curve Modeling (LGCM) will be used to test H1 and H2 using MPLUS [68]. A significant change in the slope between the baseline and experimental time periods (H1) or between Core and Enhanced conditions (H2) would suggest that the intervention affected the growth curve. This analysis will be repeated for each targeted outcome measure in the Cascade. To the extent that there are site differences, data can be analyzed within sites, using non-parametric simplified time series (STS) analysis [69, 70]. MPLUS will also allow examination of time-varying covariates to determine whether early implementation activities have significant effects on later time points.

H3a utilizes bi-weekly intake cohorts and tests whether percentages of youth meeting “timing targets” differ significantly between the 18 Enhanced and the 18 Core sites. Records data include dates to allow examination of time between various points in the Cascade (see Table 4). Trends can be examined over time using simplified time series analysis. H3b and H3c are considered exploratory, using data from agency surveys and needs assessment group interviews (measured twice: baseline and end of experiment; see Table 5). Survey content is derived from the JJ-TRIALS National Survey (developed by Chestnut Health Systems and administered in 2014). Group interviews (recorded and transcribed) generate descriptive detail on the entire Cascade, including system capacities, referral processes, the nature and use of screening instruments, the quality of available services, and features in the inner and outer contexts of agencies likely to influence service delivery.

**Table 4:** Measures from de-identified records corresponding to the Behavioral Health Service Cascade

Step	Operational definition	Rate
a. JJ referrals	Total number of referrals to juvenile justice in time period with a disposition start date, less any youth already in treatment at that time	–
b. Screened	Subset of JJ referrals (a) with a screening date	b/a
c. Clinical assessment	Subset of JJ referrals (a) with a full clinical assessment (includes if follow-up to screening or other clinical assessment)	c/a
d. Need identified	Subset of JJ referrals (a) with a need for substance use treatment based on screener, urinalysis, clinical assessment, or other sources of information	d/a
e. JJ Referrals to treatment	Subset of those in need (d), referred by the juvenile justice system to substance use treatment	e/d
f. Initiated treatment	Subset of those referred to treatment (e) who have treatment start date	f/e
g. Engaged in treatment	Subset of those who initiate treatment (f) who stay in treatment for at least 6 weeks (based on treatment discharge minus treatment start date)	g/f
h. Continuing care	Subset of those engaged in treatment (g) that are still getting treatment after 90 days (whether via retention, transfer or booster)	h/g

**Table 5:** Service cascade: crosswalk of quantitative and quality measures

	Example measures of quality		
Service cascade measure	Timing	Content indicators	Procedural indicators
Substance use screening	Days between JJ intake and screening (target: within 30 days of intake)	Use of a psychometrically sound screening instrument	Training and quality assurance provided to staff
		Use of 2 or more sources of corroborating evidence when screening	Screening results used to inform referral to full clinical assessment and/or treatment
Full clinical assessment	Days between JJ intake and assessment (target: within 30 days of intake)	Use of a psychometrically sound clinical instrument	Training and quality assurance provided to staff on administration and interpretation of assessment
		Use of DSM criteria	Assessment results used to inform placement plans
		Use of two or more sources of corroborating evidence when assessing	Qualifications of staff doing assessment
Determination of substance use-related need	Days between screen/assessment and determination of need (target: 14 days)	Determined by a psychometrically sound screening or assessment instrument	Documentation of need in record/service plan
			Communication of needs across system (e.g., judge, probation officer)
Treatment referral	Days between determined need and treatment referral (target: 14 days)	Quality of services (e.g., provider is accredited)	Treatment program contact information provided
		Match between client needs and service intensity	Active referral (e.g., phone call made; transportation provided)
Treatment initiation	Days between treatment referral and first session (target: 30 days)	Use of evidenced-based treatment approach	Documentation of first session (confirmed by service agency)
Treatment engagement	Remaining in treatment for at least 6 weeks (i.e., time from treatment intake to discharge is greater than 42 days)	Minimum education requirement of staff providing treatment services	Documentation of multiple sessions (confirmed by service agency)
		Treatment completion/discharge status	
Continued care	Receiving any treatment session after 90 days of treatment	% of youth continuing in any care	Documentation of continued attendance (confirmed by service agency)
		Number of days in continuous treatment	
	Number of continuous treatment sessions		
		Retention/dosage within each episode	
		Service intensity across episodes (increasing, decreasing)	

H4 examines staff perceptions of the value of services along the Cascade. Table 6 describes domains and sample items. Analyses will focus on change in staff responses cross-sectionally over time, using staff nested within agency. Hierarchical linear modeling (HLM) [71] will serve as the basic analysis paradigm in which Enhanced and Core sites are compared. Growth modeling may be appropriate since measures will be collected approximately every 6 months, and it is expected that the groups will be equivalent at baseline. MPLUS can be used to analyze these data using “known class” as an indicator of implementation condition in a multigroup analysis (e.g., linear growth curve modeling). Time-invariant and time-varying covariates that may differentially affect the growth curves of the two implementation conditions will be examined. Should growth model specification not fit the data, multilevel regression modeling will be used.

**Table 6:** Staff survey domains and example items

Domain	Stem and Example Items	# Items
Primary Research Question: Perceptions of Value and Use of Practices		
Substance Use Services - Resources		
Necessary Resources^a^	I have the right tools to identify substance use needs in the youth on my caseload.	9
Agency Norms^a^	My agency plays an important role in linking youth to substance use services.	4
Substance Use Services - Importance and Use	How important are the following to YOU? --	
	How often are the following USED with justice-involved youth on your Caseload? --	
Screening^b,c^	Screening youth for substance problems.	14
Assessment^b,c^	Conducting a comprehensive assessment of substance use and related problems.	18
Referral to Treatment^b,c^	Referring the youth with a substance problem to treatment services.	26
Treatment^b,c^	Encouraging every youth with a substance problem to initiate treatment services.	18
HIV/STI and Substance Use Prevention Services – Importance	How important are the following to YOU? --	
HIV/STI Prevention^b^	Educating youth about safe sex practices.	4
HIV/STI Testing^b^	Recommending that all youth be tested for HIV as part of their service plan.	7
HIV/STI Treatment^b^	Promptly linking youth with HIV seropositive test results to HIV treatment services.	8
Substance Use Prevention^b^	Strengthening youth anti-drug use attitudes, beliefs, and norms.	6
Exploratory Research Question: Implementation Process and Data Use		
Youth Data - Importance and Use [77]		
Performance Expectancy^a^	Using youth data can be helpful for -- assessing whether needs of youth are being met.	5
Social Influence^a^	The senior management of this agency promotes use of youth data to inform decisions.	2
Facilitating Conditions^a^	I have the resources necessary to use youth data to inform decisions.	3
Anxiety^a^	I feel confident about using youth data to inform decisions.	3
Intention to Use^a^	In the next 3 months -- I predict I will use youth data to inform decisions.	2
Domain	Stem and Example Items	# Items
Individual Use^d^	Within the past 3 months -- I have used youth data to assess whether needs of youth are being met.	5
Implementation Process Strategies - Importance^b^	How important is each of the following when your agency is changing a policy, process, or procedure? -- Involving an interagency workgroup that includes representatives from both juvenile justice and behavioral health providers.	10
Exploratory Research Question: Inter-agency Collaboration		
Inter-organizational Relationship [78]		
Resource Exchanges^d^	To what extent does your agency -- send results from screening youth for alcohol or drug problems to < <your partner agency> > ?	6
Resource Needs^e^	To attain your agency’s goals, to what extent does your agency need services, resources, or support from < <your partner agency> > ?	3
Challenges to Collaboration^e^	To what extent is your agency’s relationship with < <your partner agency> > hampered by -- concerns about youth confidentiality and release of information?	6
Effectiveness of Relationship^e^	To what extent does < <your partner agency> > carry out commitments it agreed to with your agency?	4
Agency and Personal Awareness^e^	To what extent -- does your agency collaborate with < <your partner agency> > in planning delivery of services to youth?	3
Quality of Communications^e^	When you wanted to communicate with persons in < <your partner agency> > during the past six months, -- how much difficulty have you had in getting in touch with them?	4
Frequency of Communications^f^	During the past six months, how frequently have you -- sent or received material (of any kind) by mail, courier, or fax with anyone in < < your partner agency> > ?	4
Exploratory Research Question: Inner Context as Moderators of Implementation Effectiveness		
Structural Characteristics of the Organization	Staff size and diversity, caseload, client census, client composition (i.e., characteristics of juveniles on supervision), accreditation, etc.	18
Organizational Climate [79]		
Innovation/Flexibility^g^	New ideas are readily accepted here.	6
Domain	Stem and Example Items	# Items
Performance feedback^g^	People usually receive feedback on the quality of work they have done.	5
Quality^g^	This organization is always looking to achieve the highest standards of quality.	4
Organizational Support^g^ [80]	This organization really cares about my well-being.	8
Organizational Functioning [81]		
Communication^a^	Staff members always feel free to ask questions and express concern.	6
Stress^a^	Staff members are under too many pressures to do their jobs effectively.	4
Satisfaction^a^	You are satisfied with your present job.	6
Adaptability^a^	You are willing to try new ideas even if some staff members are reluctant.	4
Encourages Innovation^a^	Your supervisor encourages staff to try new ways to accomplish their work.	4
Organizational Needs^a^	Your organization needs additional guidance in -- assessing youth needs.	7

Note: Scale Range: ^a^Disagree strongly, Disagree, Neither disagree nor agree, Agree, Agree strongly; ^b^Not important, Slightly important, Moderately important, Important, Very important; ^c^Not used, Some-times, Half the time, Most times, All the time; ^d^Never, Rarely, Occasionally, Frequently, Very Frequently; ^e^Not at all, A little bit, Somewhat, A fair bit, Very much; ^f^Zero times, 1 time, About 2 times, About 3 times, About every month, About every two weeks, About every week, About every 2-3 days, About every day; ^g^Definitely false, Mostly false, Mostly true, Definitely true

### Trial status

#### Feasibility testing

Feasibility testing was conducted in Spring 2015 in three sites not participating in the main study. Study protocol components tested included staff orientations, BH and goal achievement training content, data collection procedures for the needs assessment and baseline staff surveys, content and format of the site feedback report and DDDM templates, and elements of the Enhanced intervention (facilitation, LCT meetings). Information gleaned from feasibility sites was gathered in a systematic format and shared weekly with the Study Design Workgroup. As modifications to content and presentation formats were made, revised protocols were tested in other feasibility sites. Recommended modifications were reviewed and approved by the Steering Committee in September 2015. The extensive testing of all materials, trainings, and procedures in multiple sites helped ensure that anticipated variability across the 36 main study sites was accounted for and addressed.

#### Main trial

Thirty-six sites from seven states were recruited between January and December 2014. RCs began working with their six respective sites to start obtaining de-identified records in the Fall of 2014. In February 2015, sites corresponding to each RC were paired and randomized to one of three start times. After agency surveys were completed (November 2015), one site from each of the 18 pairs was randomized to the Core (*n* = 18) or Enhanced (*n* = 18) study condition. The study began in wave 1 sites in April 2015, with waves 2 and 3 beginning June and August, respectively.

## Discussion

The JJ-TRIALS protocol, developed through a collaborative partnership among NIDA, researchers, and JJ partners, has the potential to impact the field of implementation science as well as JJ and BH service systems in significant ways.

### Implementation science innovations

The engagement of JJ partners as collaborators throughout study design, implementation, and interpretation of results has been key to JJ-TRIALS. Active involvement of JJ partners in decisions is essential in designing a study that is both scientifically sound and grounded in the realities confronting the system. For JJ partners, involvement has created a sense of ownership, enhancing the likelihood that interventions are adopted and sustained.

There is great complexity in interactions between the JJ system and community service providers. The problem-solving orientation inherent in EPIS [35] is valuable in understanding the myriad factors that may affect system change: outer context issues, inner context organizational issues, and consumer concerns. These factors become the leverage points for effectively intervening to promote durable system change. EPIS is also fruitful as a framework for developing implementation strategies. The linear phases provide a platform for content and timing of intervention strategies and measurement, yet the dynamic aspect of EPIS suggests recursive movement through those phases as agencies assess and modify implementation efforts. JJ-TRIALS utilizes these strengths of EPIS and builds on current approaches to measuring process improvement [44].

DDDM is another innovative component that is compatible with the needs of researchers who rely on data for evaluating study activities and JJ partners who rely on data to demonstrate accountability to data-driven goals. Participants are trained in applying data-informed strategies using a blended learning approach [72] to facilitate the use of evidence-based practices in identifying and addressing youths’ service needs. Process mapping [73] helps identify addressable gaps in cross-systems service integration. Moreover, reliance on information already captured in sites’ service record data (both electronic and paper formats) allows tracking of the downstream changes resulting from implementation activities.

Finally, JJ-TRIALS efforts (from both quality improvement and evaluation perspectives) are aimed at the entire Cascade, from identification of need (screening and clinical assessment), linkage to care, through retention in treatment. While the JJ system has made progress in the past two decades in determining procedures for the identification of BH needs [74], far less attention has been paid to the implementation of sound procedures for addressing those needs [33]. JJ-TRIALS uses a hybrid measurement model [22] that incorporates measurement of these Cascade-related outcomes at multiple levels: systems, agencies, staff, and youth.

### Challenges and potential solutions

Several challenges inherent in developing a complex multisite protocol with multiple levels of measures and hypotheses became apparent as the JJ-TRIALS SC prepared to launch this protocol. First, to test H1, and to introduce local site leadership and staff to the basic concepts and components of the study, a baseline period was established in which data on current services and staff/organizational factors could be collected. Engaging sites in orientation and data collection activities while seeking to ensure that sites did not prematurely begin to address gaps in the Cascade presented a practical challenge.

A second challenge relates to the feasibility of implementing the complex protocol, both for the RCs and participating agencies. With six geographically separated sites per RC, simultaneously initiating the study in all sites would have presented a substantial burden that might have resulted in incomplete or poor implementation of study components. Accordingly, the design included a phased rollout (similar to a stepped wedge design) [64, 75], in which one-third of the matched site pairs were randomly assigned to begin the study in each of three waves, 2 months apart.

Another key concern reflects challenges in meeting the needs and expectations of complex, dynamic service systems while maintaining fidelity to the study protocol. Because JJ agencies face a number of competing priorities and resource constraints, RCs must be sensitive to these issues and maintain flexibility in the study timetable to maintain buy-in among stakeholders. Yet, consistent implementation across sites and across RCs is essential for internal validity. Therefore, flexibility was built into the intervention to allow for variability. Extensive fidelity procedures were developed, including pre- and post-implementation checklists for each intervention component, fidelity monitoring of trainings and facilitation, and monthly facilitator learning circle calls. Each emphasizes “fidelity with flexibility”—keeping to the written protocol to the best of the RC’s ability, while being responsive to the specific needs, preferences, and constraints of the site whenever possible.

Data quality has also proven to be a challenge. As anticipated, wide variability exists in the quality of data available to populate the Cascade. Some sites maintain electronic systems and routinely capture most Cascade elements, while others primarily utilize paper records. Even when data are available electronically, validity can be questioned (e.g., missing values could reflect absence of a service or failure to record a service). RCs have worked closely with sites to ensure adequate and appropriate data, including sending research staff to the site to manually extract records or providing assistance to JJ agencies in developing/modifying electronic systems. In this regard, JJ-TRIALS is likely to facilitate improved data collection within participating sites, addressing existing gaps in justice agencies’ ability to track and report youth outcomes [76].

### Conclusions

Through a collaborative partnership among researchers, JJ partners, and NIDA, JJ-TRIALS is incorporating several implementation strategies and the EPIS framework to address unmet substance use treatment needs among juveniles under community supervision. Although such a complex implementation study presents challenges, the protocol is expected to provide important insight regarding the efficacy of implementation interventions to improve BH services in a multi-system context, a test of the utility of EPIS for measuring and assessing organizational and systems changes, the use of a new Cascade framework for analyzing youth data on substance use services, and the ability of JJ and BH agencies to use data-driven decision making to achieve system change. Increasing the use of evidence-based practices for identifying, referring, and treating youth with substance use problems will improve both public health and public safety and provide new tools and strategies for JJ agencies and their BH partners to use when addressing other organizational and system improvements.

### Ethical approval

IRB approval was granted by all six research institutions and the coordinating center.

## Abbreviations

BH: behavioral health
CC: coordinating center
DDDM: data-driven decision-making
EBP: evidence-based practice
EPIS: Exploration, Preparation, Implementation, Sustainment framework
HIV: human immunodeficiency virus
HLM: hierarchical linear modeling
ICC: Intraclass Correlation Coefficient
IRB: institutional review board
JJ: Juvenile Justice
JJ-TRIALS: Juvenile Justice—Translational Research on Interventions for Adolescents in the Legal System
LGCM: Latent Growth Curve Modeling
LCT: local change team
NIATx: Network for the Improvement of Addiction Treatment
NIDA: National Institute on Drug Abuse
NIH: National Institutes of Health
RC: research center
SC: steering committee
STI: Sexually Transmitted Infection
STS: simplified time series
